# Adult-onset pseudohypoparathyroidism type 1B diagnosed by methylation analysis: A case report and diagnostic considerations

**DOI:** 10.1097/MD.0000000000047943

**Published:** 2026-03-13

**Authors:** Hong Sang Choi, Hee Kyung Kim, Sang Heon Suh, Chang Seong Kim, Seong Kwon Ma, Soo Wan Kim, Eun Hui Bae

**Affiliations:** a Department of Internal Medicine, Chonnam National University Medical School, Gwangju, Republic of Korea; b Department of Internal Medicine, Chonnam National University Hospital, Gwangju, Republic of Korea.

**Keywords:** case report, DNA methylation, genetic testing, hypocalcemia, mutation, pseudohypoparathyroidism

## Abstract

**Rationale:**

Pseudohypoparathyroidism type 1B (PHP1B) is a rare endocrine disorder caused by epigenetic defects at the GNAS locus, leading to isolated renal resistance to parathyroid hormone (PTH). Although typically identified in childhood, adult-onset cases are uncommon and easily overlooked. This case highlights the diagnostic challenge of late-onset PHP1B and the critical role of methylation-specific testing.

**Patient concerns:**

A 33-year-old man was referred for evaluation of incidentally detected hypocalcemia (serum calcium 5.9 mg/dL) during a routine health examination. He complained of mild paresthesia of the hands and eyelid twitching but had no family history of endocrine disorders and exhibited no phenotypic features of Albright hereditary osteodystrophy.

**Diagnoses:**

Laboratory evaluation revealed persistent hypocalcemia (serum calcium 7.7 mg/dL) and markedly elevated PTH levels (284.2 pg/mL) despite correction of magnesium and vitamin D insufficiency. Standard sequencing of the *GNAS* and *STX16* genes showed no pathogenic variants. However, methylation-specific multiplex ligation-dependent probe amplification (MS-MLPA) identified gain of methylation in the NESP55 region and loss of methylation in the AS, XL, and A/B differentially methylated regions confirming a diagnosis of sporadic PHP1B.

**Interventions:**

The patient received oral calcium carbonate and cholecalciferol supplementation. Magnesium deficiency was corrected with oral magnesium oxide.

**Outcomes:**

The patient remained asymptomatic during follow-up with adequate calcium supplementation.

**Lessons:**

Adult-onset PHP1B should be considered in the differential diagnosis of unexplained hypocalcemia with elevated PTH, even in the absence of Albright hereditary osteodystrophy. Because conventional sequencing cannot detect imprinting defects, epigenetic testing such as MS-MLPA is essential for definitive diagnosis. Increased awareness of atypical, late-onset presentations can aid in timely diagnosis and appropriate management.

## 1. Introduction

Pseudohypoparathyroidism (PHP) encompasses a group of rare endocrine disorders characterized by target-organ resistance to parathyroid hormone (PTH), resulting in hypocalcemia, hyperphosphatemia, and elevated PTH levels.^[1]^ While estimates suggest a prevalence of 0.3 to 1.1 per 100,000 population, the exact prevalence remains unknown.^[2]^ PHP is classified based on the renal response to exogenous PTH. In PHP type 1, there is a blunted increase in urinary cAMP and phosphate, whereas PHP type 2 presents with a normal cAMP response but an impaired phosphaturic effect.^[2]^ PHP type 1 is further divided into subtypes 1A, 1B, and 1C, based on genetic etiology and the presence of Albright hereditary osteodystrophy (AHO) features such as short stature, round face, and brachydactyly. PHP1A and PHP1C are associated with AHO features and multiple hormone resistance. In contrast, PHP1B is characterized by isolated renal PTH resistance and typically presents without AHO features.^[2]^

The underlying mechanism of PHP1B involves epigenetic defects at the *GNAS* locus on chromosome 20q13.2 to q13.3, which is subject to complex genomic imprinting.^[3]^ This imprinting is regulated by methylation at several differentially methylated regions (DMRs). Standard genetic sequencing is usually insufficient at identifying these changes, necessitating the use of methylation-specific multiplex ligation-dependent probe amplification (MS-MLPA) to detect imprinting defects. In addition, PHP1B most commonly presents in childhood, and adult-onset cases are relatively rare.^[2]^ Consequently, diagnosing adult-onset PHP1B can be particularly challenging. Here, we describe a case of PHP1B in an adult male, diagnosed by MS-MLPA after the incidental detection of hypocalcemia. While large-scale observational studies are vital for establishing general guidelines, focused case reports remain indispensable for delineating specific pathophysiological features and diagnostic challenges in rare and complex conditions, thereby supplementing our overall understanding of diseases like PHP1B.^[4]^ This case highlights the importance of considering PHP1B in the differential diagnosis of unexplained hypocalcemia.

## 2. Case presentation

A 33-year-old man was referred to a nephrology clinic following the incidental detection of hypocalcemia during a routine health examination, where his serum calcium was 5.9 mg/dL. He reported no medical conditions and was not taking any medications. He described symptoms of tingling in the hands and eyelid twitching. On physical examination, Chvostek sign was present. He was referred to our clinic 2 weeks later, at which time the calcium level had increased to 7.7 mg/dL after empirical oral calcium carbonate supplementation initiated by a local physician. Further laboratory testing revealed a serum ionized calcium level of 1.7 mEq/L, a phosphorus level of 4.7 mg/dL, a magnesium level of 0.78 mg/dL, a creatinine level of 0.9 mg/dL, and a PTH level of 284.2 pg/mL. His vitamin D levels were 25-hydroxyvitamin D at 27.5 ng/mL and 1,25-dihydroxyvitamin D at 28.2 pg/mL (Table 1). He exhibited no physical features suggestive of AHO, such as brachydactyly, round face, or dental anomalies.

**Table 1:** Laboratory values.

Test	Value at presentation	Normal range
Serum BUN (mg/dL)	15.8	8–23
Serum creatinine (mg/dL)	0.90	0.5–1.3
Serum sodium (mEq/L)	142	136–146
Serum potassium (mEq/L)	4.6	3.5–5.1
Serum chloride (mEq/L)	102	98–110
Serum total calcium (mg/dL)	7.7	8.4–10.2
Serum ionized calcium (mEq/L)	1.7	2.2–2.6
Serum phosphorus (mg/dL)	4.7	2.5–5.5
Serum magnesium (mg/dL)	0.78	1.1–1.5
Intact PTH (pg/mL)	284.2	15–68.3
Serum 25-hydroxyvitamin D (ng/mL)	27.5	30–50
Serum 1,25-dihydroxyvitamin D (pg/mL)	28.2	19.9–79.3

BUN = blood urea nitrogen, PTH = parathyroid hormone.

**Figure 1.**
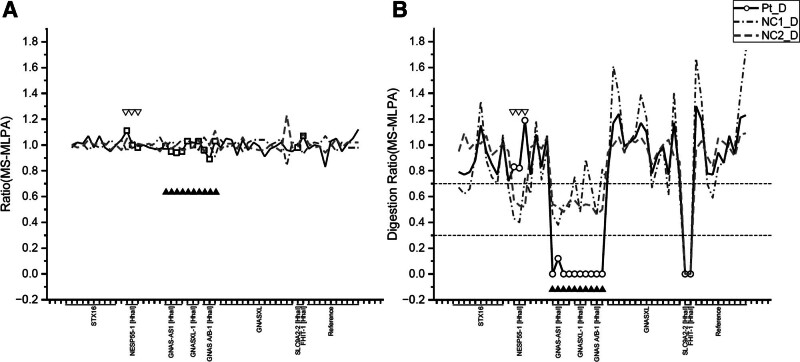
MS-MLPA analysis of the *GNAS* locus demonstrating (A) a normal copy number in the patient prior to HhaI restriction enzyme treatment, (B) an abnormal methylation pattern in the patient following HhaI restriction enzyme treatment. The methylation ratio shows gain of methylation at NESP55 (empty arrowheads) and loss of methylation at AS, XL, and A/B (solid arrowheads) in the patient compared to healthy controls (NC1 and NC2). MS-MLPA = methylation-specific multiplex ligation-dependent probe amplification.

The patient mild hypomagnesemia and borderline vitamin D insufficiency were corrected with oral magnesium oxide (400 mg/day) and cholecalciferol (1000 IU/day). Serum calcium and PTH levels remained unchanged after 4 weeks of repletion, supporting that these abnormalities were not the primary cause of hypocalcemia. Given the presence of hypocalcemia with normal 25-hydroxyvitamin D levels and elevated PTH, PTH resistance was suspected and a strong clinical suspicion of PHP was established. To investigate this diagnosis, DNA sequencing and methylation-specific multiplex ligation-dependent probe amplification (MS-MLPA) of the *GNAS* and *STX16* genes were performed. Sequencing detected no pathogenic mutations. However, MS-MLPA demonstrated abnormal methylation patterns at the *GNAS* locus following HhaI restriction enzyme treatment (Fig. 1). Specifically, the patient showed gain of methylation in the *NESP55* region and loss of methylation in the AS, XL, and A/B DMRs, consistent with a PHP1B diagnosis. As both parents had normal serum calcium levels, no further genetic testing was conducted. The patient remained asymptomatic on follow-up with adequate calcium supplementation.

## 3. Discussion

Adult-onset PHP1B is rare and poses significant diagnostic challenges. Because PHP most commonly manifests in childhood or adolescence, diagnosis in adults is often delayed due to atypical presentations.^[2]^ Unlike PHP1A, patients with PHP1B do not exhibit the characteristic AHO features, such as brachydactyly or short stature, that would otherwise serve as phenotypic clues. In their absence, hypocalcemic symptoms may be misattributed to more common etiologies, leading to diagnostic delays. These diagnostic delays are substantial, for example, a recent series of adult PHP1B patients showed a median delay of 11 years to diagnosis^[5]^, consistent with consensus guidance that PHP1B is often unrecognized until adolescence or adulthood.^[2]^ Previous reports have documented PHP cases presenting for the first time as late as the fifth decade of life.^[6]^ The present case, diagnosed at 33 years old, adds to the limited number of adult-onset PHP1B cases reported in the literature. In one series, the diagnosis had been missed for years despite long-standing symptomatic hypocalcemia, emphasizing the importance of thorough evaluation in adults with unexplained hypocalcemia and elevated PTH.^[2]^ We considered differential diagnoses of vitamin D deficiency, chronic kidney disease, hypomagnesemia, and activating mutations of the calcium-sensing receptor. Although hypomagnesemia and vitamin D insufficiency can lead to functional hypoparathyroidism or secondary hyperparathyroidism, correction of these abnormalities in our patient did not normalize serum calcium or PTH levels, thereby supporting a diagnosis of PHP. Specifically, laboratory findings that reveal a paradoxical combination of hypocalcemia, hyperphosphatemia, and elevated PTH should raise clinical suspicion for PHP1B, even in the absence of phenotypic abnormalities. Although our case showed high normal serum phosphate rather than hyperphosphatemia, similar cases have been reported, and this could have distracted the diagnosis.^[7,8]^ Additionally, if magnesium and vitamin D deficiencies exist, they should be corrected first.

Confirming the diagnosis of PHP1B requires correlating biochemical findings with molecular genetic testing. Historically, the Ellsworth-Howard test has been employed to demonstrate renal PTH resistance in PHP type 1 variants.^[9]^ In this test, a blunted rise in urinary cAMP and reduced phosphaturic response is consistent with PHP type 1, whilst an isolated phosphaturic defect with normal cAMP response is characteristic of PHP type 2. However, such biochemical testing cannot reliably distinguish between PHP1B and PHP1A, and a definitive diagnosis requires molecular confirmation.^[2]^ In PHP1B, conventional gene sequencing is often uninformative, as the condition arises from epigenetic defects at the *GNAS* locus on chromosome 20q13.3 rather than coding mutations. MS-MLPA is therefore essential for detecting the hallmark loss of imprinting at *GNAS*. Specifically, this technique can detect broad methylation changes at the *GNAS* DMRs, most notably loss of maternal exon A/B methylation, thereby confirming PHP1B.^[10,11]^ In autosomal-dominant PHP1B, these epigenetic changes are most often due to a maternally inherited deletion in the *STX16* gene, which leads to loss of methylation at exon A/B.^[10,12]^ In sporadic cases of PHP1B, no coding deletions are identified, but MS-MLPA still reveals the same epigenetic signature of PTH-resistant hypocalcemia.^[10]^ In the present case, the use of MS-MLPA was critical to identify the *GNAS* methylation defect, whereas standard sequencing would not have revealed the diagnosis. Given that PHP1B is a rare disorder with variable and often delayed presentations, case reports focusing on adult-onset and specific genotypic findings, such as those identified by MS-MLPA, remain critical for refining clinical diagnostic patterns and advancing global understanding of this complex imprinting disorder.^[13]^

Comparison with previously reported adult-onset PHP1B cases highlights both shared and distinct features of the current case. Most documented adult patients with PHP1B have presented with hypocalcemic symptoms, such as neuromuscular irritability or seizures, typically in the third to fifth decade of life.^[6]^ These neuromuscular manifestations require caution as they may be misinterpreted as nonspecific by physicians who do not have sufficient insight into rare diseases. Beyond peripheral neuromuscular irritability, chronic hypocalcemia, particularly in the context of hypoparathyroidism, is linked to central nervous system manifestations such as seizures, basal ganglia calcification, and cognitive impairment often described.^[14,15]^ As with the present case, these patients typically lacked AHO features and had no prior history of endocrine dysfunction, initially obscuring the correct diagnosis.^[12]^ For instance, Iida et al described a patient diagnosed at 46 years of age with no overt phenotypic abnormalities.^[16]^ More recently, Kostopoulos et al described a 41-year-old man with familial PHP1B due to an *STX16* deletion, identified primarily through biochemical screening rather than clinical features.^[12]^ These cases suggest that some individuals with PHP1B may remain asymptomatic into adulthood, or exhibit nonspecific symptoms, contributing to significant delays in diagnosis. In a literature review of 120 PHP1B patients, only around 38% had overt hypocalcemic symptoms and fewer than 3% displayed AHO-like features.^[12]^ This heterogeneity in clinical presentations emphasizes the need to maintain a high index of suspicion for PHP1B and to perform thorough biochemical evaluations for adult patients with unexplained hypocalcemia.

## 4. Conclusion

In conclusion, this case highlights the diagnostic challenge of adult-onset PHP1B and the critical need to consider PTH resistance in the differential diagnosis of unexplained hypocalcemia across all ages. Timely use of modern molecular diagnostics, such as MS-MLPA, is pivotal for accurate diagnosis and enables appropriate long-term management.^[17]^ Further, upon confirmation of PHP1B, genetic counseling should be recommended to assess family history, particularly for the autosomal-dominant form, and to offer targeted screening for at-risk relatives.

## Acknowledgments

This research was supported by the National Research Foundation of Korea (NRF) funded by the Korea government, MSIT (RS-2023-00217317) and the Korea Health Technology R&D Project through the Korea Health Industry Development Institute funded by the Ministry of Health and Welfare, Republic of Korea (RS-2024-00439029), and by a grant (BCRI26034) of Chonnam National University Hospital Biomedical Research Institute.

## Author contributions

**Funding acquisition:** Hong Sang Choi, Eun Hui Bae.

**Writing – original draft:** Hong Sang Choi.

**Writing – review & editing:** Hong Sang Choi, Hee Kyung Kim, Sang Heon Suh, Chang Seong Kim, Seong Kwon Ma, Soo Wan Kim, Eun Hui Bae.

**Conceptualization:** Hee Kyung Kim, Eun Hui Bae.

**Supervision:** Eun Hui Bae.
